# Psychometric performance of the Functional Assessment of Chronic Illness Therapy (FACIT) Fatigue questionnaire among adults with paroxysmal nocturnal hemoglobinuria

**DOI:** 10.1186/s41687-026-00996-4

**Published:** 2026-01-24

**Authors:** David Cella, Susan Vallow, Georgina Bermann, Jeffrey McDonald, Gilbert Ngerano, Samantha Linton, Ethan Arenson, Samopriyo Maitra, Glorian Yen, Nina Galipeau, Gavin Dickie

**Affiliations:** 1 Northwestern University, Evanston, IL USA; 2 Novartis Pharmaceutical Corporation, One Health Plaza, East Hanover, NJ 07936 USA; 3 Novartis Pharma AG, Basel, Switzerland; 4 Adelphi Values LLC, One Lincoln Street, Suite 2900, Boston, MA 02111 USA; 5 Novartis Healthcare Pvt. Ltd., Rangareddy, Hyderabad, India

**Keywords:** Paroxysmal nocturnal hemoglobinuria, PNH, FACIT-Fatigue, Dimensionality, Psychometric, MWPC

## Abstract

**Background:**

Two Phase 3 clinical trials were conducted to investigate the efficacy and safety of iptacopan, a novel treatment for paroxysmal nocturnal hemoglobinuria (PNH), a rare acquired hemolytic disorder whose most prevalent symptom is fatigue. The FACIT-Fatigue questionnaire version 4 was administered to participants to document change in patient-reported fatigue after receiving treatment with iptacopan. The aim of this study is to further evaluate the psychometric properties of the FACIT-Fatigue as a measure of fatigue among adults with PNH.

**Methodology:**

Post-hoc analyses were conducted using data from the two clinical trials at Baseline and Days 42, 126, 140, and 168. After description of its item properties (item distribution and item scores over time), the scores produced by the FACIT-Fatigue were evaluated to examine reliability (internal consistency and stability), validity (construct validity, known-groups, and responsiveness to change) and dimensionality. Meaningful within-patient change estimates were also developed.

**Results:**

Across timepoints, participants endorsed the full range of the FACIT-Fatigue’s response options, and there was monotonicity of responses showing decreasing fatigue severity over time. Dimensionality analyses supported the use of the FACIT-Fatigue total score for measurement of fatigue. The total score demonstrated very high internal consistency reliability (Cronbach’s α ≥ 0.90) and good to excellent stability (test-retest intra-class correlation ≥ 0.89), correlated with other assessment scores as expected, and was able to distinguish between clinically distinct known groups. Anchor-based analysis suggested meaningful within-patient change for the FACIT-Fatigue total score (possible score range = 0–52) to be in the range of 7.5–9.5 points.

**Conclusions:**

The psychometric results reported here provide evidence suggesting that the scores produced by the FACIT-Fatigue are reliable and valid, and thus may be considered fit for purpose in measuring fatigue among adults with PNH. While unidimensional confirmatory factor analysis did not entirely reflecting a unidimensional model, use of a bifactor model did provide evidence of essential unidimensionality, supporting the conclusion that FACIT-Fatigue total score may be appropriate to use in evaluating treatment efficacy in this target patient population.

**Supplementary Information:**

The online version contains supplementary material available at 10.1186/s41687-026-00996-4.

## Background

In the context of clinical trials, the use of patient-reported outcome (PRO) measures is considered the gold standard in collecting patient experience data and may be considered vital in demonstrating the value and efficacy of novel treatments from the patient perspective [1–5]. This is particularly true when collecting data on experiences that can only be reported by the patients themselves, such as pain or fatigue.

Two Phase 3 clinical trials (APPLY-PNH and APPOINT-PNH) were conducted to investigate the efficacy and safety of iptacopan, a novel treatment for paroxysmal nocturnal hemoglobinuria (PNH). PNH is a rare acquired hemolytic disorder characterized by complement-mediated intravascular hemolysis, bone marrow failure, anemia, and severe thrombophilia [6]. Concomitant fatigue is the most prevalent symptom of the condition [7–9].

To assess this critical symptom in PNH, a PRO instrument designed to measure the symptoms and impacts of fatigue, the Functional Assessment of Chronic Illness Therapy – Fatigue (FACIT-Fatigue) version 4, was administered to participants in APPLY-PNH and APPOINT-PNH to document change in patient-reported fatigue after receiving treatment with iptacopan. The FACIT-Fatigue has been used in other recent clinical trials [10–12], and it has been included in the US FDA Clinical Outcome Assessment (COA) Compendium as an acceptable measure for assessing fatigue in PNH [13].

The content validity of the FACIT-Fatigue has been documented in cognitive debriefing interviews with patients with PNH, demonstrating that the PRO instrument is interpretable and that the concepts of fatigue assessed by the measure are relevant to the patient experience of PNH [9]. At present there is little available evidence to demonstrate the reliability and validity of scores produced by the FACIT-Fatigue when measuring the fatigue experience of individuals with PNH. The research presented here reflects a post-hoc analysis of data from the APPLY-PNH and APPOINT-PNH clinical trials. The objectives of this research were to address this evidence gap and in line with PRO measurement best practice [1], report that the FACIT-Fatigue demonstrates acceptable psychometric properties, and that, within the available analysis samples, the FACIT-Fatigue total score based on a model of a single latent factor is appropriate for the measurement of fatigue in adults with PNH.

## Methods

### Trials and analysis populations

APPLY-PNH (NCT0482053019) was a multicenter, randomized, open-label, active-comparator-controlled study comparing adult participants who were being treated with a C5 inhibitor and experiencing residual anemia, and were randomly assigned to either continue their previous C5 inhibitor or switch to iptacopan. APPOINT-PNH (NCT0455891820) was a multicenter, single-arm, open-label trial with adult participants who had never received complement inhibitor therapy and received iptacopan during the study [14]. Across the two trials participants were recruited from 15 countries including: Brazil, China, Czech Republic, France, Germany, Italy, Japan, Malaysia, Netherlands, Singapore, South Korea, Spain, Taiwan, UK, US. See Table 1 for further information on each of these clinical studies.

**Table 1:** Description of clinical trials used as data sources for analysis

Clinical trial	Sample size	Study design	Treatment groups	Key eligibility criteria
APPLY-PNH	*N* = 97 adult participants	Multicenter, randomized, open-label, active comparator-controlled, parallel group study comprising a screening period followed by a 24-week, active-controlled, parallel-group treatment period, followed by a 24-week iptacopan treatment extension period for all patients	Participants were randomized into one of two treatment arms in an 8:5 ratio: •200 mg of iptacopan monotherapy twice daily •IV anti-C5 antibody treatment	•Adult participants ≥18 years of age with a diagnosis of PNH •Stable regimen of anti-C5 antibody treatment for at least 6 months prior to randomization, but still presenting with residual anemia •Mean hemoglobin level < 10 g/dL •Participants could not be on a stable eculizumab regimen with a dosing interval of 11 days or less, or on a stable ravulizumab regimen with a dosing interval of less than 8 weeks; and could not have a known or suspected hereditary complement deficiency at screening; history of hematopoietic stem cell transplantation; laboratory evidence of bone marrow failure; active system bacterial, viral, or fungal infection within 14 days prior to study drug administration; history of recurrent invasive infections; or any major concurrent comorbidities
APPOINT-PNH	*N* = 40 adult participants	Multicenter, single-arm, open-label trial comprising a screening period (up to 8 weeks) followed by a 24-week core treatment period and a 24-week extension treatment period	•200 mg of iptacopan twice daily	•Adult participants ≥18 years of age with a diagnosis of PNH •Mean hemoglobin level < 10 g/dL •LDH > 1.5 × upper limit of normal for at least two central laboratory measurements two to eight weeks apart during the screening period •Participants could not be on prior treatment with a complement inhibitor; known or suspected hereditary complement deficiency at screening; history of hematopoietic stem cell transplantation; laboratory evidence of bone marrow failure, active system bacterial, viral, or fungal infection within 14 days prior to study drug administration; history of recurrent invasive infections; or any major concurrent comorbidities

Abbreviations: IV = intravenous; LDH = lactate dehydrogenase; PNH = paroxysmal nocturnal hemoglobinuria

Three psychometric analysis samples were defined: the first included participants from APPLY-PNH (the APPLY sample), and the second included participants from APPOINT-PNH (the APPOINT sample). Both samples included all participants with non-missing FACIT-Fatigue scores at Baseline and at least one follow-up timepoint. A third analysis sample including participants from both clinical trials (the Pooled sample) was created to provide a sample size expected to be large enough for to evaluate dimensionality and to make dimensionality analysis as robust as possible. Because both the APPLY-PNH and APPOINT-PNH trials reported a total score for the FACIT-Fatigue, the relationships between scores produced by the questionnaire’s items were examined to test whether they are explained well enough by a single domain to justify reporting a total score; this is what is referred to as dimensionality analysis [15, 16]. The Pooled sample was built upon the assumption that with respect to dimensionality the APPLY and the APPOINT samples would both behave similarly (as supported by consistent reliability and validity results for both samples). All psychometric analyses except for the FACIT-Fatigue dimensionality analysis were conducted separately for the APPLY and APPOINT samples.

In addition, two test-retest (TRT) analysis samples were defined to include participants from the APPLY-PNH clinical trial (APPLY-TRT) and the APPOINT-PNH clinical trial (APPOINT-TRT) who demonstrated stability in their condition, defined as no more than two weeks elapsed between Screening and Day 1 and a stable health status in the absence of any systematic intervening effects between those timepoints, as required to evaluate test-retest reliability. Evaluation of test-retest reliability for the FACIT-Fatigue was conducted separately for each analysis sample. The analysis samples are described in Table 2.

**Table 2:** Definitions of analysis samples

Psychometric analysis samples	Test-retest analysis samples
**The APPLY sample:** All Full Analysis Set (FAS) participants from APPLY-PNH clinical trial with non-missing FACIT-Fatigue scores at Baseline and at least one follow-up timepoint (Day 42, Day 126, Day 140, and/or Day 168).	**APPLY-TRT:** APPLY sample participants who have: The same PGIS-F responses at screening and Day 1 No more than two weeks elapsed between screening and Day 1 No transfusion between Screening and Day 1 Stable Hgb between screening and Day 1
**The APPOINT sample:** All FAS participants from APPOINT-PNH clinical trial with non-missing FACIT-Fatigue scores at Baseline and at least one follow-up timepoint (Day 42, Day 126, Day 140, and/or Day 168).	**APPOINT-TRT:** APPOINT sample participants who have: The same PGIS-F responses at Screening and Day 1 No more than two weeks elapsed between screening and Day 1 No transfusion between Screening and Day 1 Stable Hgb between Screening and Day 1
**The Pooled sample:** All pooled data from the APPLY and APPOINT samples at Week 24.	Not applicable

Abbreviations: FACIT-Fatigue = Functional Assessment of Chronic Illness Therapy – Fatigue; FAS = Full Analysis Set; Hgb = hemoglobin; PGIS-F = Patient Global Impression of Severity of Fatigue; TRT = Test-retest analysis sample

### Assessments

The FACIT-Fatigue version 4, the target instrument for the psychometric evaluation reported here, is a 13-item PRO questionnaire developed to assess the fatigue experience and fatigue impact of adult respondents (18 years and older) within a reflective indicator model. It uses a five-point verbal rating scale ranging from “Not at all” to “Very much” and a recall period of “the past 7 days.” A total score for the FACIT-Fatigue is calculated by summing the item scores, after reversing item scores that are worded in the negative direction (i.e., Items 1–6 and 9–13). The total score ranges from 0 to 52, where a higher score indicates lower fatigue severity [17]; FACIT-Fatigue mean scores in the general population have been found to be in the range of 43.5 points [18].

To support the evaluation of the psychometric performance of the FACIT-Fatigue, three supplemental assessments were included in the analyses: the European Organization for The Research and Treatment of Cancer Quality of Life Questionnaire (EORTC QLQ-C30) [19, 20] physical function, role function, fatigue, dyspnea, and global health status domains; the Five-level EQ-5D (EQ-5D-5 L) [21] mobility, self-care, usual activities, and pain/discomfort domains; and the Patient Global Impression of Severity of Fatigue (PGIS-F) [22]. Please see Supplementary Table 1 for further information about the supplemental assessments.

The FACIT-Fatigue and supplemental assessments were completed by participants using an electronic device, and participants received training on using the device. Assessments were completed in the native language of the participants using validated translations.

### Analyses

The analyses for psychometric evaluation were performed to evaluate the scores produced by the FACIT-Fatigue in terms of reliability, validity, and dimensionality. Analyses were pre-specified in a Psychometric Statistical Analysis Plan and were conducted with SAS Version 9.4 (SAS Institute, Cary, North Carolina) [23]. Analysis timepoints were selected to be consistent with all other analyses in the clinical trials, which sought to assess persistence of effect by evaluating scores throughout the last four study visits (rather than at a single timepoint). Table 3 summarizes the analyses and criteria used for this psychometric evaluation of the FACIT-Fatigue, and each analysis is explained in further detail below.

**Table 3:** Summary of psychometric evaluation of FACIT-Fatigue

Property type	Tests/analyses	Analysis samples	Timepoints	Assessment criteria
Item properties	Item distribution	APPLY sample APPOINT sample	• Baseline • Day 42 • Day 126 • Day 140 • Day 168	Item scores over time
Dimensionality	Inter-item correlations	Pooled sample	• Day 140 • Day 168	Spearman’s |*r*|≥0.30 [24]
	Item-scale analysis			Spearman’s |*r*|≥0.30
	Unidimensional CFA			RMSEA closer to 0.00
	Bifactor model			PUC < 80.0% and high PECV, and Mean relative bias < 15.0%
Total score reliability	Internal consistency reliability	APPLY sample APPOINT sample	• Baseline • Day 42 • Day 126 • Day 140 • Day 168	Cronbach’s α ≥ 0.70 McDonald’s ω≥0.70
	Test–retest reliability	APPLY-TRT APPOINT-TRT	• Baseline • Day 1	ICC≥0.50 [25, 26]
Construct-related validity	Concurrent validity	APPLY sample APPOINT sample	• Baseline • Day 42 • Day 126 • Day 140 • Day 168	Spearman’s |*r*|≥0.30 Polyserial
	Known-groups	APPLY sample APPOINT sample	• Baseline • Day 168	Means increase with greater severity 95% CI does not overlap with adjacent groups
	Responsiveness to change	APPLY sample APPOINT sample	• Baseline • Day 42 • Day 168	Spearman’s |*r*|≥0.30
Score interpretation	MWPC	APPLY sample	• Day 126 • Day 140 • Day 154 • Day 168	Improvement on FACIT-Fatigue total score corresponding to 1-level improvement on PGIS-F

Abbreviations: CFA = confirmatory factor analysis; CI = confidence interval; ICC = intra-class correlation; MWPC = meaningful within-person change; PECV = percentage explained common variance; PGIS-F = Patient Global Impression of Severity of Fatigue; PUC = percentage of uncontaminated correlation; RMSEA = root-mean-square error of approximation; TRT = Test-retest analysis sample

*Item properties* were examined by describing the distribution of responses to the FACIT-Fatigue across trial timepoints.

*Dimensionality:* To evaluate treatment efficacy in APPLY-PNH and APPOINT-PNH, the FACIT-Fatigue was assumed to have a unidimensional structure and was therefore scored to create a total score. Previous studies (one in chronic lymphocytic leukemia and another in cancer), while identifying two subscales in the FACIT-Fatigue, also demonstrated sufficient unidimensionality of the total scale; therefore, the FACIT-Fatigue can be reliably scored as a single value [27, 28]. To confirm the unidimensionality of the FACIT-Fatigue in this population, dimensionality analyses were conducted using the Pooled sample at Day 140 and Day 168, the last two timepoints at which FACIT-Fatigue scores were collected from participants. While Baseline assessments are typically used for initial psychometric evaluation, there is increasing recognition of the need to evaluate scale performance at later timepoints in a clinical trial – particularly the final two post-treatment assessments [29–31]. These timepoints were selected to provide evidence of stability of the dimensionality analyses towards the end of the trials. Dimensionality analyses included:

- Inter-item correlations: In a unidimensional structure, it is expected that items should be at least moderately correlated (|*r*| > 0.30) with each other; consistently weak correlations may indicate that items may measure different concepts [24, 32].
- Item-scale analysis: In a unidimensional structure, it is expected that items should be correlated not only with each other but also with the larger scale score. For the FACIT-Fatigue, to remove bias, correlations were examined between items and the corrected total score (defined as the total FACIT-Fatigue score not including the score of the item being correlated).
- Unidimensional confirmatory factor analysis (CFA): Conducted to provide an indicator of how different the unidimensional model-predicted item correlations were from the observed item correlations. Analysis was conducted by examining root-mean-square error of approximation (RMSEA). Common interpretative guidelines suggest that RMSEA values closer to 0.00 indicate better fit of items in a unidimensional model. An RMSEA ≥0.80 suggests poor fit [15, 16, 33].
- Bifactor model: The bifactor model is based on two assumptions: all items are explained by one common domain, and each item is explained by exactly one “grouping factor” (the part of the correlations that the common domain cannot explain). In this analysis, two grouping factors were hypothesized [28]: experience items (feeling listless, feeling tired, having energy, feeling weak all over, and feeling fatigued) and impact items (too tired to eat, needing help with daily activities, frustration, limitation to social activities, trouble starting things, trouble finishing things, ability to do usual activities, and needing to sleep during the day). If the percentage of uncontaminated correlation (PUC) is ≥80.0%, (or if the PUC is < 80.0%, and the percentage explained common variance [PECV] is high, indicating that a large percentage of item variability can be explained by a general domain), and the mean relative bias (which measures the discrepancy of the standardized factor loadings from the unidimensional model in relation to those from the bifactor model [15]) is < 15.0% in magnitude, this is considered sufficient evidence of essential unidimensionality to support reporting the FACIT-Fatigue total score [34].

*Reliability of the FACIT-Fatigue total score:* Reliability estimates characterize consistency and reproducibility of a particular set of scores produced by an instrument when administered to a target patient population and in a specific context of use [35]. Reliability of the FACIT-Fatigue total score was evaluated using the following methods:

- Internal consistency reliability: Internal consistency describes how the items within a single domain correlate with each other. In reporting a total score for the FACIT-Fatigue, one would expect all items to demonstrate strong internal consistency. The internal consistency of the FACIT-Fatigue was investigated by calculating (1) Cronbach’s alpha coefficient (α) [36], which is a lower bound to internal consistency, and (2) McDonald’s omega coefficient (ω) [37]. Both coefficients range from 0 to 1, with higher estimates indicative of stronger internal consistency among items [38, 39]. While there are no universally accepted rules for the interpretation of α, estimates > 0.70 are typically seen as sufficient for research purposes [39].
- Test-retest reliability: Test-retest reliability for the FACIT-Fatigue total score was evaluated for each participant as the intra-class coefficient (ICC) for absolute agreement in a two-way mixed-effects model (in which time was a fixed effect and participants were a random effect) [26]. ICC values range from 0 to 1, with higher values indicating better reliability; values < 0.50 generally indicate poor reliability while values > 0.90 generally indicate excellent reliability.

*Construct-related validity of the FACIT-Fatigue total score:* Validity relates to the extent to which an instrument conforms to logical relationships that should exist with measures of related concepts [1]. Using the total score, FACIT-Fatigue construct-related validity was examined to demonstrate that the FACIT-Fatigue total score was (1) correlated with other assessments as expected (concurrent validity analysis), (2) able to distinguish between clinically distinct subgroups (known-groups analysis), and (3) responsive to change.

- Concurrent validity: FACIT-Fatigue total scores were compared to scores of items or domains in three supplementary assessments: the EORTC QLQ-C30 (physical function, role function, fatigue, dyspnea, and global health status domains), the EQ-5D-5 L (mobility, self-care, usual activities, and pain/discomfort domains), and the PGIS-F (overall severity of PNH-related fatigue). The strength and direction of the relationships were hypothesized a priori and expected to be stronger for concepts that are similar in nature (i.e., convergent validity or evidence) and weaker for more distal concepts (i.e., discriminant or “divergent” validity or evidence). Comparisons were made for both the APPLY sample and the APPOINT sample at Baseline and Days 42, 126, 140, and 168. For each supplementary assessment and timepoint, the appropriate correlation was computed; as a rule of thumb, a correlation of ≥|0.30| was considered sufficient evidence of concurrent validity.
- Known-groups analysis: For the FACIT-Fatigue total score, known groups were evaluated using the PGIS-F to define pre-specified three groups based on response (No symptoms/Mild, Moderate, and Severe/Very Severe) at Baseline and Day 168. Nonparametric ANOVA was used to calculate confidence intervals.
- Responsiveness to change: The correlations between changes in the FACIT-Fatigue total score and changes in the EORTC QLQ-C30 fatigue symptom scale and the PGIS-F were examined between Baseline and Day 42 and between Baseline and Day 168 for each analysis sample. Negative correlations were expected due to the opposite scoring directions of the instruments: higher FACIT-Fatigue total score indicates lower fatigue burden, while higher scores on the EORTC QLQ-C30 fatigue symptom scale and the PGIS-F indicate greater fatigue burden. It was hypothesized that the FACIT-Fatigue total score would exhibit moderate negative correlations to both.

*Meaningful within-person change (MWPC):* In its recent Patient Focused Drug Development guidance documents, the United States Food and Drug Administration reiterates previous guidance on the need to document that changes in COA scores are meaningful to patients [1, 5]. One method for doing this is to estimate MWPC (the difference in a COA score on an individual patient level that would be meaningful to that patient). The MWPC for the FACIT-Fatigue was estimated using data from APPLY-PNH and a one-level change in severity category on the PGIS-F as anchor. A one-level change was selected as anchor because, in qualitative interviews conducted with a subset of participants in APPLY-PNH [40], the majority indicated that a one level change on the PGIS-F would be meaningful to them. Score changes from Baseline were examined at Days 126, 140, 154, and 168 of the trial.

*Missing data:* Participants may have had data missing for a variety of reasons, including non-compliance, premature study discontinuation, administrative issues, or a missed study visit. For this psychometric evaluation study, missing data were not imputed for any of the measures. For the APPLY sample (*N* = 95), there was no missing data at Baseline, one participant had missing data at Day 42, six participants had missing data at Day 126, four participants had missing data at Day 140, and three participants had missing data at Day 168. For the APPOINT sample (*N* = 40), there was no missing data at Baseline and Day 42, one participant had missing data at Day 126, two participants had missing data at Day 140, and three participants had missing data at Day 168.

## Results

### Description of analysis samples

The APPLY sample consisted of 95 adult participants (two participants had insufficient data for analysis and so could not be included in the APPLY sample) in APPLY-PNH. Ages ranged from 20 to 84 years (mean = 51.4 years, standard deviation [SD] = 16.8), and more than half (*n* = 65, 68.4%) of the sample were male (see Table 4). Most were in Germany (*n* = 20, 21.1%), Italy (*n* = 17, 17.9%), and France (*n* = 15, 15.8%). The APPOINT sample consisted of 40 adult participants in APPOINT-PNH. Ages ranged from 18 to 84 (mean = 42.1, SD = 15.8). Most were in China (*n* = 20, 50.0%) followed by France, Germany, and the United Kingdom (each with *n* = 4, 10.0%).

**Table 4:** Demographic information at Baseline for psychometric analysis samples

Characteristic	APPLY sample Statistic or n (%)	APPOINT sample Statistic or n (%)
**Age (years)**		
N	95	40
Mean (SD)	51.4 (16.8)	42.1 (15.8)
Median	53.0	38.5
Min, max	20.0, 84.0	18.0, 81.0
Sex		
Male	65 (68.4%)	23 (57.5%)
Female	30 (31.6%)	17 (42.5%)
**Country of residence (n, %)**		
Brazil	4 (4.2%)	No enrollment
China	No enrollment	20 (50.0%)
Czech Republic	1 (1.1%)	No enrollment
France	15 (15.8%)	4 (10.0%)
Germany	20 (21.1%)	4 (10.0%)
Italy	17 (17.9%)	2 (5.0%)
Japan	9 (9.5%)	No enrollment
Malaysia	No enrollment	3 (7.5%)
Netherlands	4 (4.2%)	No enrollment
Singapore	No enrollment	1 (2.5%)
South Korea	1 (1.1%)	2 (5.0%)
Spain	4 (4.2%)	No enrollment
Taiwan	3 (3.2%)	No enrollment
UK	9 (9.5%)	4 (10.0%)
US	8 (8.4%)	No enrollment

### Item properties of the FACIT-Fatigue

Item distribution for both the APPLY and APPOINT samples were examined at Baseline and Days 42, 126, 140, and 168. Across these timepoints, participants endorsed the full range of the FACIT-Fatigue’s response options. Floor effects were observed for 10 items In the APPLY sample and for 11 items in the APPOINT sample, while no ceiling effects were observed in the APPLY sample, and ceiling effects were observed for one item in the APPOINT sample. In both analysis samples, there was monotonicity of responses showing an increase in score (and thus a decrease in severity of fatigue) over time.

### Dimensionality of the FACIT-Fatigue

The Pooled sample, used for dimensionality analyses, consisted of 128 participants at Day 140 and 131 participants at Day 168. At both timepoints, inter-item correlations ranged from moderate (|*r*| > 0.38) to very strong (|*r*| > 0.96), and item-scale correlations ranged from moderate (|*r*| > 0.56) to very strong (|*r*| > 0.89). In unidimensional CFA, the RMSEA indicated a marginal fit at Day 140 (RMSEA = 0.09), which improved to an acceptable level by Day 168 (RMSEA = 0.08). In bifactor model analysis, at Day 140, while PUC was 67.2%, PECV was high at 98.2%, and relative bias was ≤5.1% in magnitude; at Day 168, while PUC was also 67.2%, PECV was high at 97.1% and relative bias was ≤8.2% in magnitude, providing evidence of essential unidimensionality to support reporting the FACIT-Fatigue total score [15, 16].

### Reliability of the FACIT-Fatigue total score

FACIT-Fatigue scores demonstrated very high internal consistency at Baseline and Days 42, 126, 140, and 168 in both the APPLY and APPOINT samples, with estimates of α consistently above 0.89; although ω could not be estimated at two timepoints because of lack of convergence of the factor analysis model, estimates of ω at all other timepoints were consistently above 0.90, supporting the very high internal consistency of the FACIT-Fatigue scores. See Table 5 for results.

**Table 5:** Internal consistency reliability of the FACIT-Fatigue

Timepoint	APPLY sample (*N* = 95)		APPOINT sample (*N* = 40)	
	Cronbach’s α	McDonald’s ω	Cronbach’s α	McDonald’s ω
Baseline	0.95	0.91	0.94	N/A^*^
Day 42	0.95	0.91	0.92	0.92
Day 126	0.95	N/A^*^	0.94	0.93
Day 140	0.96	0.92	0.92	0.91
Day 168	0.96	0.93	0.90	0.91

McDonald’s ω could not be estimated because the factor analysis model used to derive the estimate did not converge

Test-retest reliability was evaluated using scores at Baseline and Day 1 from APPLY-TRT and APPOINT-TRT. The ICC for APPLY-TRT was 0.934, and for APPOINT-TRT it was 0.891 (see Table 6 for results).

**Table 6:** Test-retest reliability of the FACIT-Fatigue total score

Analysis sample	n^*^	ICC	95% confidence interval	
			Lower	Upper
APPLY-TRT (*N* = 95)	16	0.93	0.82	0.98
APPOINT-TRT (*N* = 40)	19	0.89	0.74	0.96

Abbreviation: TRT = Test-retest analysis sample

Only participants with non-missing study instrument scores at both screening and Day 1, stable hemoglobin levels in the absence of transfusion, and ≤2 weeks between screening and Day 1 were included in the analysis. Missing data are due to either > 2 weeks between timepoints, missing instrument scores, or reception of transfusion or change in hemoglobin levels between timepoints

### Construct-related validity of the FACIT-Fatigue total score

Concurrent validity results demonstrated a pattern of relationships between the FACIT-Fatigue and supplementary assessments as expected for both analysis samples and across timepoints. The strongest relationships were observed with supplementary assessments measuring similar concepts as the FACIT-Fatigue, including the EORTC QLQ-C30 Fatigue (|r|= > −0.78) and PGIS-F (|r|= > −0.67), whereas correlations were weakest among assessments measuring concepts more distal to fatigue, including the EQ-5D-5 L dyspnea (|r|= > −0.10), self-care (|r|= > −0.23), and pain/discomfort (|r|= > −0.24). As expected, the FACIT-Fatigue total score was moderately to strongly correlated (|r|>±0.30) with all other supplementary assessment scores. Please see Supplementary Table 2 for complete results.

Known groups were evaluated using the PGIS-F to define groups based on response at Baseline and Day 168. PGI-F responses were collapsed into three groups (No symptoms/Mild, Moderate, and Severe/Very Severe) to ensure sufficient sample size in each group. At both Baseline and Day 168, participants in both the APPLY and APPOINT samples in the “No symptoms/Mild” group had significantly higher (better) mean and median FACIT-Fatigue total scores than participants in the “Moderate” group, who in turn had higher (better) mean and median FACIT-Fatigue total scores than participants in the “Severe/Very severe” group (see Table 7). While there was some overlap in 95% confidence interval (CI) for adjacent categories at Baseline for both analysis samples, at Day 168 no overlap was observed.

**Table 7:** Known-groups analysis for FACIT-Fatigue total score by PGIS-F response

Analysis sample	Timepoint	Collapsed PGIS-F category	n	FACIT-Fatigue total score	
				Mean (SD)	Median (95% CI)
APPLY sample	Baseline (*n* = 95)	No symptoms/mild	44	42.3 (6.9)	43.5 (40.0, 47.0)
		Moderate	37	28.5 (6.0)	27.0 (25.0, 31.0)
		Severe/very severe	14	20.2 (11.9)	22.5 (11.0, 29.0)
	Day 168 (*n* = 90)	No symptoms/mild	67	44.0 (6.9)	45.0 (43.0, 47.0)
		Moderate	17	27.9 (7.4)	29.0 (26.0, 32.0)
		Severe/very severe	6	16.7 (8.9)	19.0 (0.0, 24.0)
APPOINT sample	Baseline (*n* = 40)	No symptoms/mild	16	40.1 (7.3)	40.5 (32.0, 48.0)
		Moderate	18	29.7 (8.2)	28.5 (23.0, 36.0)
		Severe/very severe	6	20.5 (8.5)	17.5 (13.0, 35.0)
	Day 168 (*n* = 37)	No symptoms/mild	33	45.2 (5.2)	46.0 (45.0, 48.0)
		Moderate	4	33.5 (3.7)	34.0 (29.0, 37.0)
		Severe/very severe	0	N.A. (N.A.)	N.A. (N.A., N.A.)

Abbreviations: CI = confidence interval; FACIT-Fatigue = Functional Assessment of Chronic Illness Therapy – Fatigue; PGIS-F = Patient Global Impression of Severity of Fatigue; SD = standard deviation

For responsiveness-to-change analysis, change scores on the EORTC QLQ-C30 fatigue symptom scale showed strong to very strong correlations with the changes on the FACIT-Fatigue total score. For the APPLY sample at Day 42, mean change in the FACIT-Fatigue total score was 5.39, yielding correlations of *r* = −0.74 (95% CI [−0.82, −0.63]); at Day 168, mean change in the FACIT-Fatigue total score was 5.82, yielding correlations of *r* = 0.76 (95% CI [0.84, 0.66]). For the APPOINT sample at Day 42, mean change in the FACIT-Fatigue total score was 8.97, yielding correlations of *r* = −0.78 (95% CI [−0.88, −0.61]); at Day 168, mean change in the FACIT-Fatigue total score was 12.38, yielding correlations of *r* = 0.78 (95% CI [0.88, 0.60]).

Similar results were seen with the PGIS-F. At Day 42, correlations were *r* = −0.81 (95% CI [−0.87, 0.72]) for the APPLY sample, and *r* = −0.74 (95% CI [−0.86, −0.56]) for the APPOINT sample. At Day 168, correlations were *r* = 0.74 (95% CI [0.82, 0.63]) for the APPLY sample and *r* = 0.83 (95% CI [0.91, 0.69]) for the APPOINT sample.

### FACIT-Fatigue total score meaningful within-patient change

**Fig. 1:**
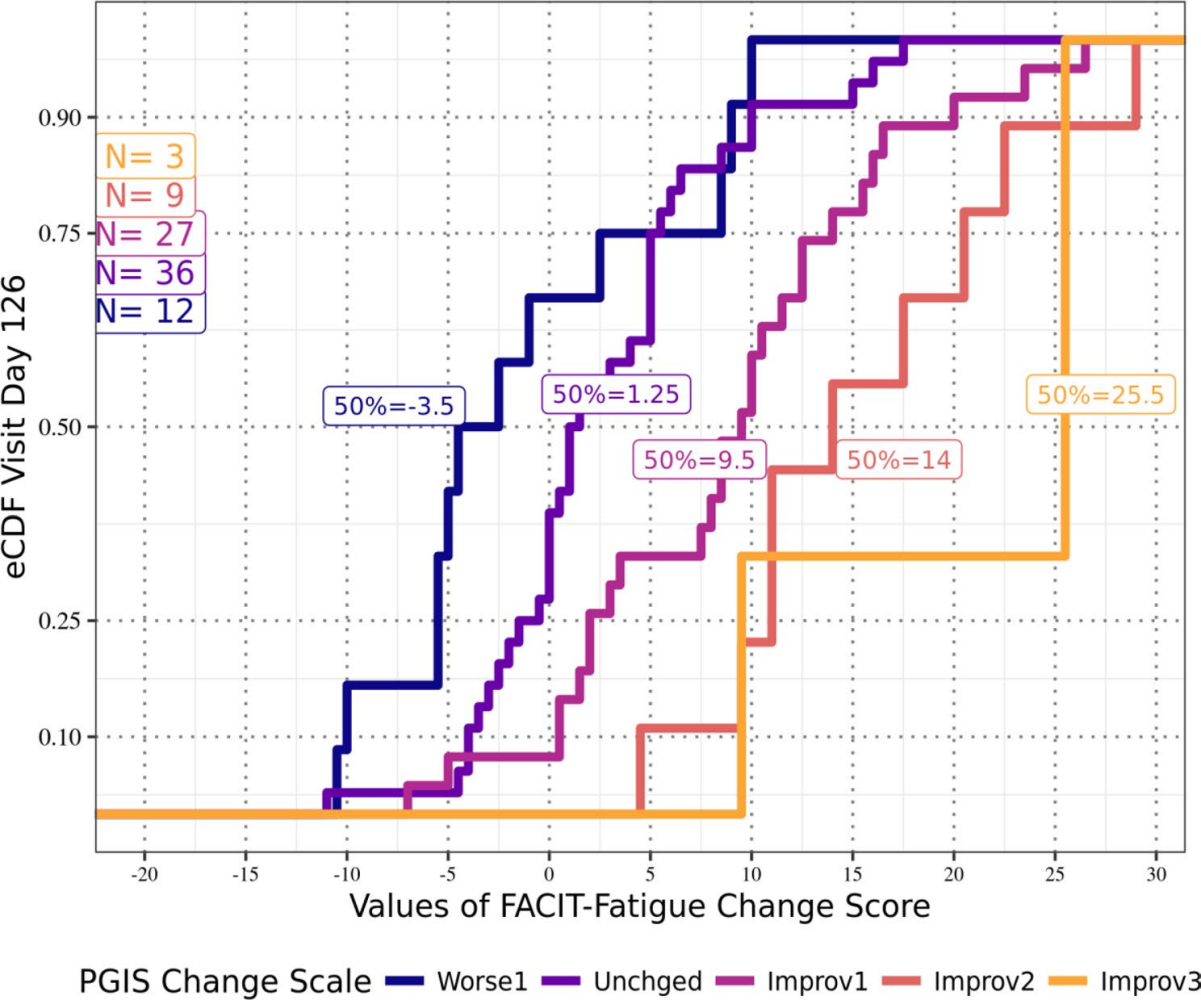
eCDF of FACIT-Fatigue change score values by PGIS-F change categories Day 126 (*N* = 87)

**Fig. 2:**
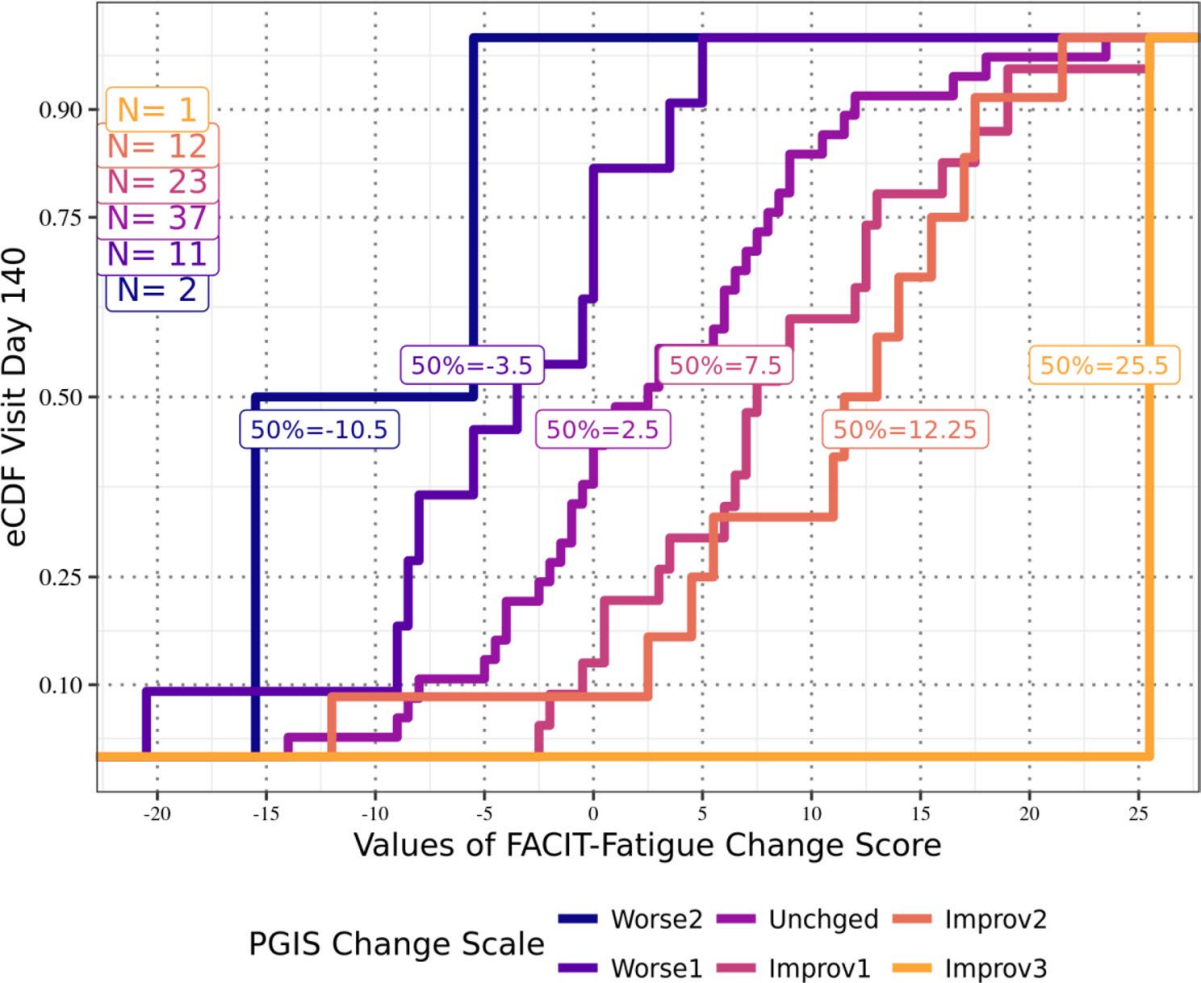
eCDF of FACIT-Fatigue change score values by PGIS-F change categories Day 140 (*N* = 86)

**Fig. 3:**
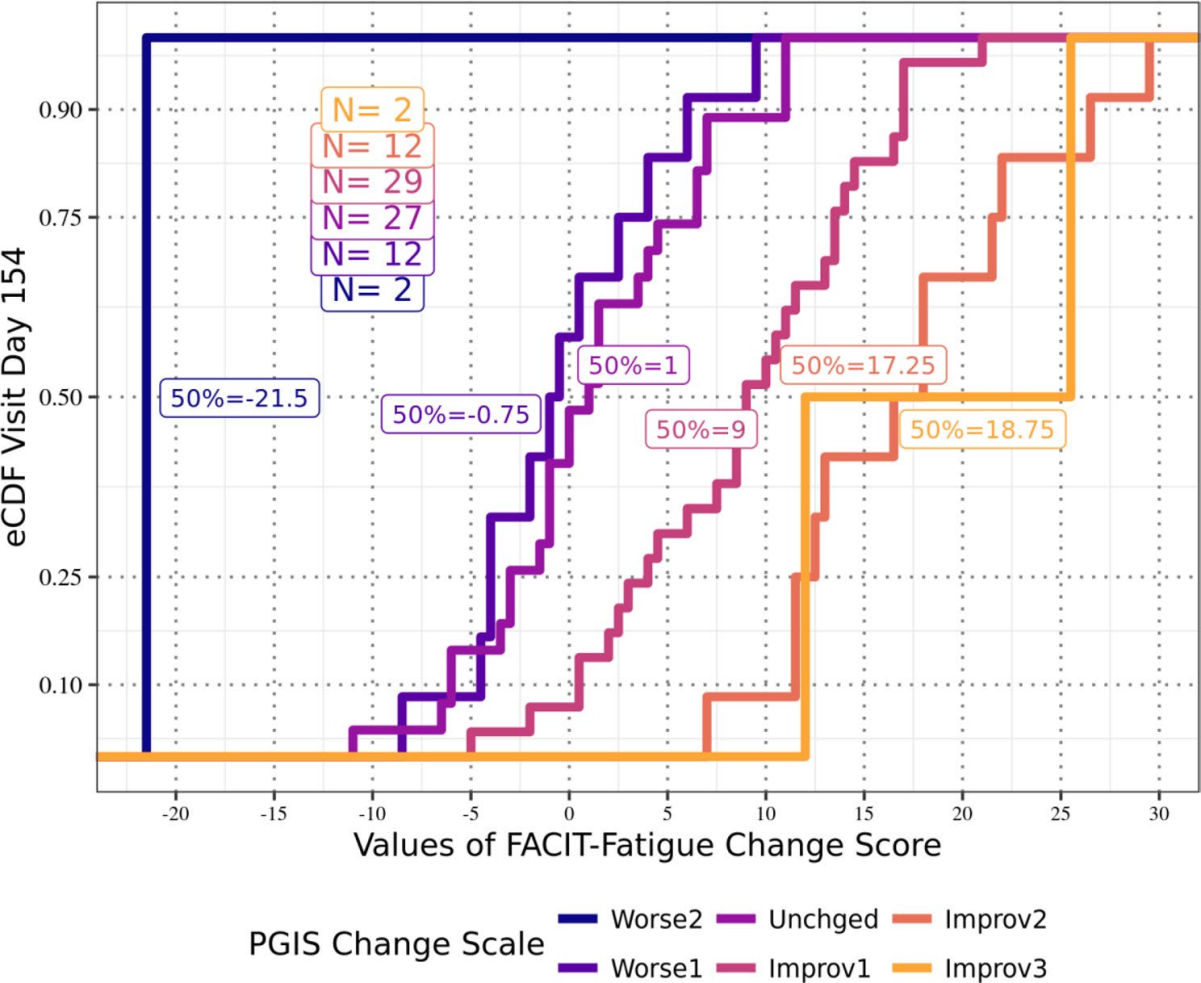
eCDF of FACIT-Fatigue change score values by PGIS-F change categories Day 154 (*N* = 84)

**Fig. 4:**
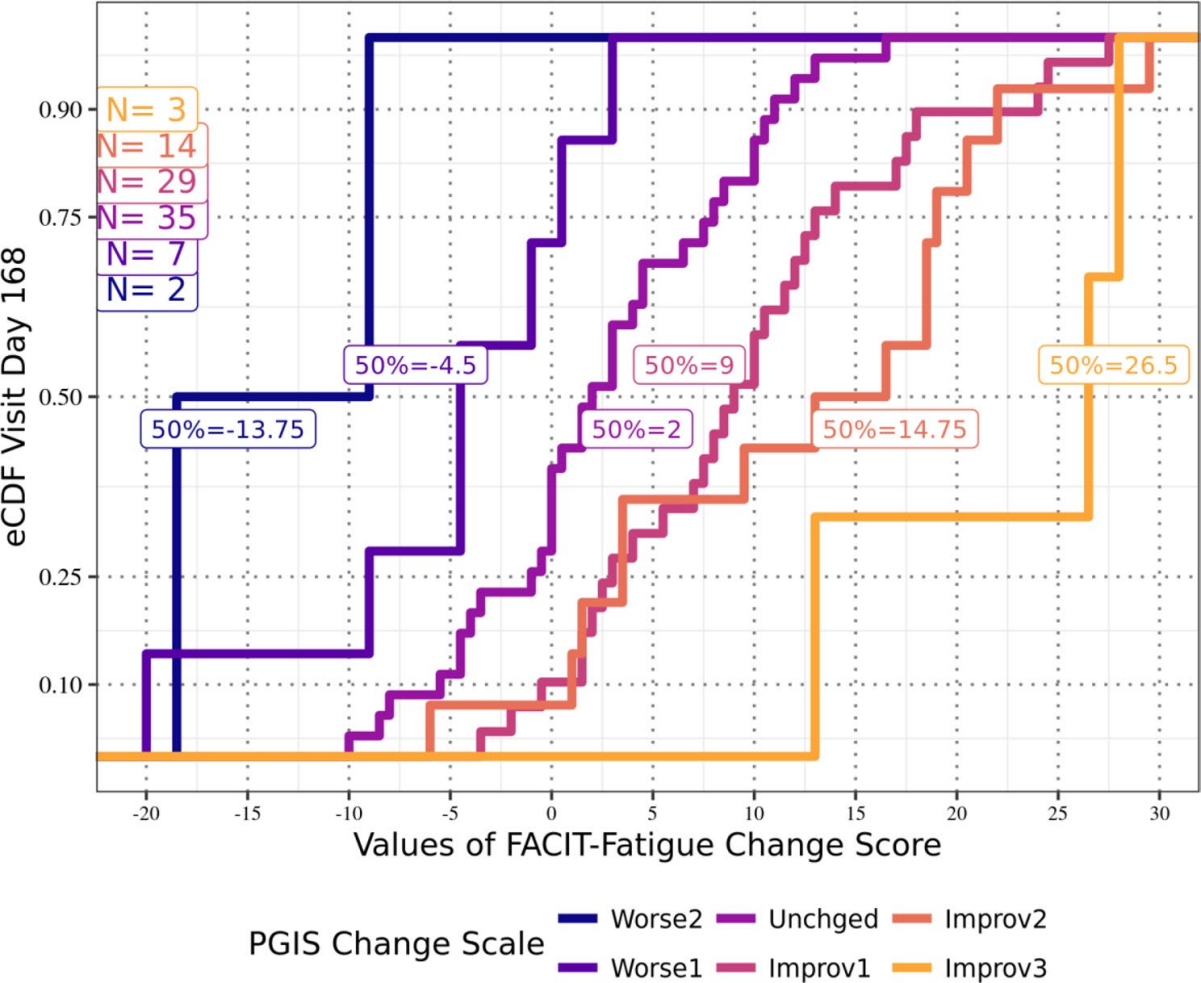
eCDF of FACIT-Fatigue change score values by PGIS-F change categories Day 168 (*N* = 90)

Anchor-based analyses of the FACIT-Fatigue total scores for the APPLY sample showed that at Day 126 (Fig. 1), participants reporting a one-level improvement in PGIS-F also reported a median improvement from Baseline of 9.5 points on the FACIT-Fatigue total score; at Day 140 (Fig. 2), those participants reported a median improvement of 7.5 points; and at both Day 154 (Fig. 3) and Day 168 (Fig. 4), those participants reported a median improvement of 9.0 points. These results suggest an MWPC for the FACIT-Fatigue to be in the range of 7.5–9.5 points.

In Figs. 1–4, **Worse2** reflects a 2-point worsening in PGIS-F score from Baseline; **Worse1** reflects a 1-point worsening in PGIS-F score from Baseline; **Unchged** indicates the PGIS-F score did not change from Baseline; **Improv1** reflects a 1-point improvement in PGIS-F score from Baseline; **Improv2** reflects a 2-point improvement in PGIS-F score from Baseline; and **Improv3** reflects a 3-point improvement in PGIS-F score from Baseline.

## Discussion

This study describes the psychometric properties of the FACIT-Fatigue in adult participants with PNH across the APPLY-PNH and APPOINT-PNH clinical trials, in order to provide support for the appropriateness of the questionnaire in assessing fatigue related symptoms and impacts in PNH. Findings from the present analysis demonstrate that scores from the FACIT-Fatigue are reliable and valid in PNH, and add further empirical evidence supporting the use of the FACIT-Fatigue in PNH adult patient populations [22, 41].

In both clinical trials, there was evidence that participants used the full range of response options. There was still a monotonicity of responses showing decrease in severity of fatigue over time, which was expected in the context of effective treatment benefit.

Dimensionality analyses provide support for a single-factor structure and the use of the FACIT-Fatigue total score in this population. Inter-item correlations and item-scale correlations ranged from moderate to very strong, suggesting that FACIT-Fatigue items are closely related, as would be expected in a single-factor structure. While CFA results for a unidimensional model were inconclusive due to model fit, bifactor model analysis provided evidence of essential unidimensionality for the FACIT-Fatigue, supporting the reporting of the FACIT-Fatigue total score in this trial population.

In evaluation of its reliability, the FACIT-Fatigue total score demonstrated very high internal consistency reliability and good to excellent test-retest reliability across timepoints. These results provide evidence that the FACIT-Fatigue produces reliable total scores in this PNH trial population.

Concurrent validity results for the FACIT-Fatigue total score demonstrated relationships to supplementary assessments as expected for both populations at all timepoints, with strongest relationships observed for assessments measuring similar concepts, and weakest correlations for assessments measuring concepts more distal to fatigue. The FACIT-Fatigue total score was also able to distinguish between groups based upon collapsed PGIS-F categories (No symptoms/Mild, Moderate, Severe/Very severe). This is consistent with results reported previously [42] demonstrating that the FACIT-Fatigue total score was able to distinguish between participants in APPLY-PNH and APPOINT-PNH who had achieved an increase of ≥ 2 g/dL in hemoglobin in the absence of transfusion and those who did not, providing evidence of validity in terms of known groups.

Anchor-based analysis suggests an MWPC for the FACIT-Fatigue total score to be in the range of 7.5–9.5 points for participants in the APPLY-PNH study. This is higher than some previously reported values in PNH populations [41].

It should be noted that both APPLY-PNH and APPOINT-PNH were “open label” studies, in which participants were not blinded as to the treatment they were receiving. Additionally, the small size of the analysis samples (particularly in APPOINT-PNH and the test-retest reliability samples) could potentially limit the robustness of the analyses reported above.

Despite these limitations, the dimensionality and psychometric performance of the FACIT-Fatigue were evaluated at multiple timepoints across two clinical trials, with consistent results in both. The performance of the FACIT-Fatigue total score was evaluated not only using other PRO measures but also using objectively measured hematological endpoints. These considerations strengthen confidence in the conclusions drawn from these analyses.

## Conclusion

The psychometric results reported for the FACIT-Fatigue here provide evidence that the scores produced by the FACIT-Fatigue are reliable and valid, and thus fit for purpose in measuring fatigue among adults with PNH. Dimensionality analysis, while not entirely reflecting a unidimensional model, did provide evidence of essential unidimensionality using a bifactor model, lending support to the conclusion that FACIT-Fatigue total score may be appropriate to use in evaluating treatment efficacy in this target patient population. Additionally, anchor-based analysis suggests a change of 7.5–9.5 points as meaningful in this patient population. Further analyses using data from other PNH clinical trials, including larger sample sizes and participants from additional countries, can serve to confirm the generalizability of these findings, overcome some of the statistical constraints of the present study due to limited sample size, and provide further support that the FACIT-Fatigue is a fit for purpose tool in measuring the fatigue experience of individuals with PNH.

## Electronic supplementary material

Below is the link to the electronic supplementary material.

Supplementary Material 1

## Data Availability

The data that support the findings of this study are not publicly available due to reasons of sensitivity but are available from the corresponding author upon reasonable request.

## Abbreviations

CFA: Confirmatory factor analysis
CI: Confidence interval
COA: Clinical outcome assessment
EORTC QLQ-C30: European Organization for The Research and Treatment of Cancer Quality of Life Questionnaire
EQ-5D-5 L: Five-level EQ-5D
FACIT-Fatigue: Functional Assessment of Chronic Illness Therapy – Fatigue
ICC: Intra-class correlation
MWPC: Meaningful within-patient change
PECV: Percentage explained common variance
PGIS-F: Patient Global Impression of Severity of Fatigue
PNH: Paroxysmal nocturnal hemoglobinuria
PRO: Patient-reported outcome
PUC: Percentage of uncontaminated correlation
RMSEA: Root-mean-square error of approximation
TRT: Test-retest analysis sample
