# Supplementary material for: Psychometric performance of the Functional Assessment of Chronic Illness Therapy (FACIT) Fatigue questionnaire among adults with paroxysmal nocturnal hemoglobinuria
Source: J Patient Rep Outcomes. 2026 Jan 24;10:27. doi: 10.1186/s41687-026-00996-4 (PMC12913856; doi:10.1186/s41687-026-00996-4)
Supplement: Supplementary file 1 — Supplementary Material 1 [file 41687_2026_996_MOESM1_ESM.docx]

# Supplementary materials

| Supplementary Table 1. Supplemental assessments | | | |
| --- | --- | --- | --- |
| Assessment | Dimensions included in analysis | Scoring |  |
| EORTC QLQ-C30  *Widely used and recognized PRO questionnaire for assessing health-related quality of life, function, and symptoms* | - Physical function scale   - Five items   - Recall period: present time - Role function scale   - Two items   - Recall period: the past week - Fatigue symptom scale   - Three items   - Recall period: the past week - Dyspnea symptom item   - One item   - Recall period: the past week - Global health status (GHS)   - Two items   - Recall period: the past week | - All items except GHS employ a 4-point response scale from 1-Not at all to 4-Very much - GHS items employ a 7-point numerical rating scale from 1-Very poor to 7-Excellent - For all scales and symptom items, a raw score is produced by estimating average of all items in the scale. Raw scores are standardized using linear transformation so that scores range from 0 to 100 - Higher scores on GHS and function scales indicate better functioning and quality of life - Higher scores on symptom scales and items indicate greater symptom severity |  |
| EQ-5D-5L  *PRO questionnaire to assess symptoms, quality of life, and overall health* | - Mobility dimension - Self-care dimension - Usual activities dimension - Pain/discomfort dimension - Overall health visual analog scale (VAS) - All items use recall period of today | - Dimensions are scored on 5 levels ranging from no problem (Level 1) to unable to/extreme problems (Level 5) - VAS is scored on a scale from 0 to 100 - Higher dimension scores indicate worse health state for that dimension - Higher VAS score indicates better health state |  |
| PGIS-F  *Single item measuring severity of fatigue symptoms* | - Recall period of the past 7 days | - Employs a 5-point verbal response scale ranging from “No symptoms” (scored as 0) to “Very severe” (scored as 5) - Higher scores indicate greater fatigue symptom severity |  |

Abbreviations: EORTC QLQ-C30=European Organization for The Research and Treatment of Cancer Quality of Life Questionnaire; EQ-5D-5L=5-level EQ-5D; PGIS-F=Patient Global Impression of Severity of Fatigue

| Supplementary Table 2. Correlational analysis of the FACIT-Fatigue total score | | | | | | | | | | |
| --- | --- | --- | --- | --- | --- | --- | --- | --- | --- | --- |
| Supportive questionnaire item (score)/Clinical trial | Baseline | | Day 42 | | Day 126 | | Day 140 | | Day 168 | |
|  | n | Corr. | n | Corr. | n | Corr. | n | Corr. | n | Corr. |
| The APPLY sample | | | | | | | | | | |
| EORTC QLQ-C30: Physical function (0–‍100)^*^ | 95 | 0.76^[a]^ | 93 | 0.79^[a]^ | 87 | 0.79^[a]^ | 87 | 0.79^[a]^ | 90 | 0.75^[a]^ |
| EORTC QLQ-C30: Role function (0–‍100)^*^ | 95 | 0.75^[a]^ | 93 | 0.82^[a]^ | 87 | 0.79^[a]^ | 87 | 0.81^[a]^ | 90 | 0.83^[a]^ |
| EORTC QLQ-C30: Fatigue (0–100)^*^ | 95 | -0.82^[a]^ | 93 | -0.88^[a]^ | 87 | -0.87^[a]^ | 87 | -0.89^[a]^ | 90 | -0.83^[a]^ |
| EORTC QLQ-C30: Dyspnea (0–100)^*^ | 95 | -0.62^[a]^ | 93 | -0.57^[a]^ | 87 | -0.61^[a]^ | 87 | -0.67^[a]^ | 90 | -0.58^[a]^ |
| EORTC QLQ-C30: Global health status (0–100)^*^ | 95 | 0.71 | 93 | 0.82^[a]^ | 87 | 0.75^[a]^ | 87 | 0.83^[a]^ | 90 | 0.80^[a]^ |
| EQ-5D-5L: Mobility^†^ | 95 | -0.64^[a]^ | 93 | -0.67^[a]^ | 87 | -0.56^[a]^ | 87 | -0.66^[a]^ | 90 | -0.66^[a]^ |
| EQ-5D-5L: Self-care^†^ | 95 | -0.38^[a]^ | 93 | -0.41^[a]^ | 87 | -0.23^[a]^ | 87 | -0.41^[a]^ | 90 | -0.39^[a]^ |
| EQ-5D-5L: Usual activities^†^ | 95 | -0.74^[a]^ | 93 | -0.84^[a]^ | 87 | -0.75^[a]^ | 87 | -0.76^[a]^ | 90 | -0.72^[a]^ |
| EQ-5D-5L: Pain/‌discomfort^†^ | 95 | -0.55^[a]^ | 93 | -0.54^[a]^ | 87 | -0.27^[a]^ | 87 | -0.41^[a]^ | 90 | -0.41^[a]^ |
| EQ-5D-5L: VAS (0–100)^†^ | 95 | 0.66^[a]^ | 93 | 0.76^[a]^ | 87 | 0.73^[a]^ | 87 | 0.76^[a]^ | 90 | 0.75^[a]^ |
| PGIS (0–4)^‡^ | 95 | -0.85^[b]^ | 93 | -0.94^[b]^ | 87 | -0.89^[b]^ | 86 | -0.86^[b]^ | 90 | -0.88^[b]^ |
| The APPOINT sample | | | | | | | | | | |
| EORTC QLQ-C30: Physical function (0–‍100)^*^ | 40 | 0.81^[a]^ | 39 | 0.85^[a]^ | 35 | 0.80^[a]^ | 37 | 0.79^[a]^ | 37 | 0.71^[a]^ |
| EORTC QLQ-C30: Role function (0–‍100)^*^ | 40 | 0.82^[a]^ | 39 | 0.74^[a]^ | 35 | 0.81^[a]^ | 37 | 0.80^[a]^ | 37 | 0.75^[a]^ |
| EORTC QLQ-C30: Fatigue (0–100)^*^ | 40 | -0.83^[a]^ | 39 | -0.87^[a]^ | 35 | -0.83^[a]^ | 37 | -0.78^[a]^ | 37 | -0.82^[a]^ |
| EORTC QLQ-C30: Dyspnea (0–100)^*^ | 40 | -0.62^[a]^ | 39 | -0.47^[a]^ | 35 | -0.55^[a]^ | 37 | -0.54^[a]^ | 37 | -0.10^[a]^ |
| EORTC QLQ-C30: Global health status (0–100)^*^ | 40 | 0.57^[a]^ | 39 | 0.75^[a]^ | 35 | 0.67^[a]^ | 37 | 0.67^[a]^ | 37 | 0.53^[a]^ |
| EQ-5D-5L: Mobility^†^ | 40 | -0.45^[a]^ | 39 | -0.49^[a]^ | 35 | -0.58^[a]^ | 37 | -0.52^[a]^ | 37 | -0.46^[a]^ |
| EQ-5D-5L: Self-care^†^ | 40 | -0.43^[a]^ | 39 | -0.32^[a]^ | 35 | -0.27^[a]^ | 37 | -0.26^[a]^ | 37 | -0.28^[a]^ |
| EQ-5D-5L: Usual activities^†^ | 40 | -0.64^[a]^ | 39 | -0.64^[a]^ | 35 | -0.73^[a]^ | 37 | -0.74^[a]^ | 37 | -0.44^[a]^ |
| EQ-5D-5L: Pain/‌discomfort^†^ | 40 | -0.38^[a]^ | 39 | -0.36^[a]^ | 35 | -0.38^[a]^ | 37 | -0.36^[a]^ | 37 | -0.24^[a]^ |
| EQ-5D-5L: VAS (0–100)^†^ | 40 | 0.71^[a]^ | 39 | 0.63^[a]^ | 35 | 0.66^[a]^ | 37 | 0.59^[a]^ | 37 | 0.41^[a]^ |
| PGIS (0–4)^‡^ | 40 | -0.76^[b]^ | 39 | -0.84^[b]^ | 35 | -0.83^[b]^ | 37 | -0.74^[b]^ | 37 | -0.67^[b]^ |

Abbreviations: Corr.=correlation; EORTC QLQ-C30=European Organisation for Research and Treatment of Cancer Core Quality of Life Questionnaire; EQ-5D-5L=Five-level EQ-5D; FACIT-Fatigue=Functional Assessment of Chronic Illness Therapy – Fatigue; FAS=full analysis set; PGIS=Patient Global Impression of Severity; PsAP=psychometric analysis population; VAS=visual analogue scale

Note: The APPLY sample consists of FAS patients enrolled in the APPLY-PHN clinical trial with at least one non-missing score on the FACIT-Fatigue at Baseline and at least one follow-up timepoint. The APPOINT sample consists of FAS patients enrolled in the APPOINT-PHN clinical trial with at least one non-missing score on the FACIT-Fatigue at Baseline and at least one follow-up timepoint.

Note: Correlations are represented by ^[a]^Spearman or ^[b]^polyserial correlation coefficients.

Note: The FACIT-Fatigue total score ranges from 0 to 52 with higher scores indicating lower fatigue severity.

^*^EORTC QLQ-C30 scores range from 0 to 100. Higher scores on the physical functioning, role function, and global health status/‌quality of life scores indicate higher functioning and quality of life. Higher scores on the symptom domains indicate worse symptom experiences; for EORTC QLQ-C30 Fatigue and Dyspnea domains, higher scores indicate greater severity.

^†^The EQ-5D-5L domain items are five-response ordinal items where higher scores represent worse health states. The EQ-5D-5L VAS ranges from 0 to 100 where higher scores represent better health states.

^‡^The PGIS asks participants to rate their overall symptoms of fatigue during the past seven days. The PGIS is rated on an ordinal scale ranging from 0 to 4 with higher scores indicating greater symptom severity.
